# Mitochondria and polycystic ovary syndrome: The role of ferroptosis, inflammasomes, and endoplasmic reticulum stress

**DOI:** 10.7150/ijbs.128537

**Published:** 2026-05-01

**Authors:** Xinyi Zhang, Tong Sun, Xinxin Wang, Yujiu Ma, Liu Cao, Jichun Tan

**Affiliations:** 1 Centre of Reproductive Medicine, Department of Obstetrics and Gynaecology, Shengjing Hospital of China Medical University, Shenyang, Liaoning, China.; 2 Key Laboratory of Reproductive Dysfunction Disease and Fertility Remodelling of Liaoning Province, Shenyang, Liaoning, China.; 3 Department of Pediatrics, Shengjing Hospital of China Medical University, Shenyang, Liaoning, China.; 4 Department of Reproductive Medicine, Shenyang Maternity and Child Health Hospital, Shenyang, Liaoning, China.; 5 Clinical Translational Research Center, Shengjing Hospital of China Medical University, Shenyang, Liaoning, China.

**Keywords:** mitochondria, ferroptosis, inflammasome, endoplasmic reticulum stress, polycystic ovary syndrome

## Abstract

Polycystic ovary syndrome (PCOS) poses a major threat to women of reproductive age and is strongly associated with metabolic and inflammatory abnormalities. Over the past decade, tremendous progress has been made in our understanding of signaling events regulated by mitochondria. Emerging evidence underscores mitochondrial dysfunction as a central pathophysiological hub in PCOS. The intricate crosstalk among mitochondrial dysfunction, ferroptosis, inflammasomes, and endoplasmic reticulum (ER) stress creates a pathological network that underpins ovarian dysfunction, metabolic abnormalities, and chronic inflammation in PCOS, highlighting promising novel targets for diagnosis and therapeutic intervention in this complex disorder.

## 1. Introduction

Polycystic ovary syndrome (PCOS) is the most common reproductive and endocrine disorder affecting between 10% and 13% of women, based on the population under investigation and the diagnostic standards used 1, 2. Its primary characteristics include chronic ovulatory dysfunction, hyperandrogenism (HA), and polycystic ovarian morphology, with clinical manifestations such as menstrual irregularities, acne, hirsutism, and infertility 3. Importantly, PCOS is not merely a reproductive disorder. The majority of women with PCOS exhibit metabolic disturbances, including insulin resistance (IR), hyperinsulinemia, dyslipidemia, overweight/obesity, impaired glucose tolerance, and an elevated risk of type 2 diabetes 4-6. The pathogenesis of PCOS is complex and influenced by genetic, environmental, and hormonal factors. PCOS is associated with a higher risk of adverse pregnancy outcomes, including miscarriage, gestational diabetes, and hypertensive disorders of pregnancy 7, 8. Later in life, women with PCOS face significantly elevated risks of cardiovascular disease and endometrial cancer 9, 10. Given the complexity of PCOS and its lifelong health implications, current treatment mainly relies on symptom-based management and long-term health monitoring 11, 12.

Early diagnosis and treatment of PCOS continue to pose challenges. Several potential biomarkers of PCOS have been reported, spanning hormonal, metabolic, oxidative stress, inflammatory, and microRNA-related categories 13. Nevertheless, due to the heterogeneity of the disease, emerging evidence suggests that biomarker signatures may vary across PCOS phenotypes, and many women experience delayed diagnosis 14. Lifestyle modification remains the cornerstone of PCOS management. In addition, oral contraceptives are commonly used for menstrual irregularities and hyperandrogenism. Insulin sensitizers such as metformin are used to manage metabolic disturbances in PCOS. For infertility, letrozole is recommended as the first-line ovulation induction agent, with clomiphene citrate, gonadotrophins, and laparoscopic ovarian surgery as alternative options 15, 16. Nevertheless, there remains a dearth of more precise and effective therapeutic agents. Thus, elucidating the pathogenesis of PCOS is crucial for reducing its incidence and improving clinical outcomes.

Mitochondria, as key regulators of cellular metabolism, proliferation, aging, and programmed cell death, have emerged as a focal point in PCOS pathogenesis research, attracting increasing attention in recent years 17, 18. Furthermore, mitochondria can influence the incidence and progression of PCOS by modulating ferroptosis, inflammasome activation, and ER stress via multiple pathways, including reactive oxygen species (ROS) generation and alterations in mitochondrial metabolism 19-21. The above findings have established the role of mitochondria in PCOS. Therefore, we integrate the current literature on the complex interplay between mitochondrial damage, ferroptosis, inflammasomes, and ER stress in the molecular pathogenesis of PCOS.

## 2. PCOS and mitochondria pathophysiology

### 2.1 Mitochondrial ROS

The mainly documented mitochondrial dysfunction in PCOS pathogenesis is the elevated production of mitochondrial ROS. Mitochondria are the main sources of intracellular ROS. During normal aerobic respiration, the electron transport chain (ETC) transfers electrons to molecular oxygen for water production. However, a minor proportion of electrons leak directly to oxygen, forming superoxide anions (O^2-^), a type of ROS. O^2-^ can be further transformed by superoxide dismutase (SOD) into hydrogen peroxide (H_2_O_2_); H_2_O_2_ is relatively stable, has high membrane permeability, and can diffuse within the cell and participate in signal transduction 22. ROS are involved in the modulation of various molecules and signaling pathways, including the activation of protein kinase signaling cascades, the regulation of transcription factor activity, and calcium ion homeostasis 23, 24. Under specific pathological conditions such as mitochondrial dysfunction, hypoxia, or inflammation, pronounced electron leakage occurs within the ETC, leading to substantial ROS generation. Moreover, ROS production represents a critical mechanism of immune defense that promotes the release of chemokines and cytokines from immune cells, thereby enhancing the immune response 25.

HA and IR are the primary pathophysiological mechanisms of PCOS. Furthermore, HA elevates the risk of IR, and both are closely associated with the onset of oxidative stress, which are significant factors contributing to oxidative stress in PCOS. Research has demonstrated that PCOS patients with the HA phenotype have more severely impaired antioxidant function 26. In these patients, HA induces the expression of ovarian aldose reductase, leading to an increase in the flux of the polyol pathway, and also increases ovarian lipid peroxidation, reduces catalase activity and glutathione (GSH) content, further damaging ovarian function 27. Lower mitochondrial efficiency and increased level of mitochondrial H_2_O_2_ emissions in obese women with IR compared with lean women have been reported in a clinical randomized controlled trial. Furthermore, the level of mitochondrial H_2_O_2_ emissions in the obese group decreased after exercise 28. Similarly, the production of ROS increases in women with PCOS, accompanied by a reduction in GSH levels and oxygen consumption, and may be alleviated by the administration of metformin, a natural antioxidant 29, 30. Excessive oxidation reactions and the weakening of antioxidant capacity may be key factors that accelerate the onset of PCOS. An animal study indicated that pregnant PCOS rats exhibited a mismatch between oxidative and antioxidative stress reactions in the gravid uterus 31. Results of a study by Zhang et al. also support this conclusion, reporting that the mitochondria-ROS-SOD1/Nrf2 pathway in the placenta of PCOS rats is directly linked to the detrimental effects of IR and HA on embryonic survival. SOD1 is an important protein involved in oxidative stress, while Nrf2 participates in antioxidant responses 32. Nrf2-Foxo1-ROS and ROS/p38/JNK pathways have also been reported in PCOS models 33, 34. Oxidative stress, as a key pathological response of the body to metabolic imbalance, exhibits a significantly abnormal activation state in the ovarian tissues of PCOS patients. Its interaction with IR and HA may may form a vicious cycle that drives disease progression. In addition to direct damage, ROS also plays a significant role in mitochondrial metabolism and quality control processes 35.

### 2.2 Mitochondrial metabolism

Mitochondria serve as energy-producing organelles 36. The tricarboxylic acid (TCA) cycle and oxidative phosphorylation occur within the mitochondria and produce adenosine triphosphate (ATP). Mitochondria also act as central intermediaries connecting glucose, fatty acid, and amino acid metabolism. Pyruvate is converted into acetyl-CoA in mitochondria to participate in the TCA cycle, while β-oxidation breaks down fatty acids into acetyl-CoA, thereby supporting the TCA cycle and energy generation. Metabolic disturbances can result in the reprogramming of mitochondrial energy metabolism 37. PCOS has been found to be associated with abnormalities in mitochondrial energy metabolism, with studies mainly focusing on ovarian granulosa cells and showing decreased ATP production and reduced glycolysis levels 38, 39. Increased levels of ROS and lower expression of glycolysis-associated genes, including glucose transporter-1 (GLUT1), phosphofructokinase (PFK), and lactate dehydrogenase A (LDHA), have been reported in granulosa cells of PCOS women compared with non-PCOS women, indicating that the low oocyte competence in PCOS may be associated with mitochondrial dysfunction and abnormal glycolysis 40. A clinical study by Mazloomi et al. also found abnormalities in ATP content and glucose metabolism disorders in granulosa cells of PCOS patients. Women with PCOS show lower ATP concentrations and decreased expression levels of key glycolytic enzymes, including PFK and hexokinase 1 (HK1), accompanied by a lower glycolysis rate compared with a control group 41. In addition to reduced ATP synthesis, increased mitochondrial ROS, and decreased glycolysis, Zhang et al. verified insufficient mitochondrial oxidative phosphorylation levels in granulosa cells of PCOS women. These changes were found to be regulated by Sirtuin 3 (SIRT3) signaling in KGN cells 42. Cao et al. found that follicular fluid-derived exosomal miR-155-5p and miR-143-3p participate in the regulation of glycolysis and apoptosis of granulosa cells in the PCOS model 43. However, in ovarian stromal cells of a PCOS model, glycolysis may actually increase, but this is more related to promoting the inflammation and fibrosis than to directly supporting follicle development 44.

### 2.3 Mitochondrial quality control

#### 2.3.1 Mitochondrial biogenesis

The maintenance of mitochondrial homeostasis is governed mainly by mitochondrial quality control (MQC), which involves mitochondrial biogenesis, dynamics, and autophagy. The process of mitochondrial biogenesis maintains the quantity of mitochondria and replaces aging or damaged mitochondria with new, healthy ones. This process is rigorously regulated by the peroxisome proliferator-activated receptor gamma coactivator (PGC-1α) through the activation of Nrf1/2 and mitochondrial transcription factor A (TFAM) 45, 46. The transcription and replication of mitochondrial DNA (mtDNA) are facilitated by signaling molecules such as TFAM, which is subsequently translated into proteins and assembled into functional mitochondria. Mitochondria possess their own genetic material, mtDNA, which encodes various molecules essential for mitochondrial function. Mutations in the mtDNA can lead to mitochondrial dysfunction and diseases 47.

A randomized, triple-blind, placebo-controlled clinical trial revealed that granulosa cells of PCOS women have lower expression of important genes involved in mitochondrial biogenesis, such as TFAM and PGC-1α. This expression level may be increased by resveratrol administration, which activates Sirtuin 1 (SIRT1) and improves the mtDNA copy number 48. In a dehydroepiandrosterone (DHEA)-induced PCOS mouse model, Safaei et al. found that vitamin D3 improved mitochondrial biogenesis by stimulating the mitogen-activated protein kinase (MAPK) pathway in granulosa cells 49. Furthermore, Pang et al. reported the role of SIRT3/Foxo1/PGC-1α in KGN cells of a PCOS model, and Sun et al. showed that mitochondrial biogenesis could also be regulated by circadian clock genes REV-ERBs 50, 51. Reduced mitochondrial biosynthesis leads to decreased activity of antioxidant enzymes (such as SOD and GPX), resulting in the ROS accumulation 52. Excessive ROS further damages mtDNA and membrane structures, ultimately accelerating follicular closure 53.

mtDNA copy number and variants are also important factors in PCOS. According to Tharayil et al., women with PCOS had lower mtDNA copy numbers and also had mtDNA variations. PCOS is strongly associated with mtDNA variations such as 1488T, 9670G, 12556G, 3308G, 9200G, 15914T, 14480G, and 5426G 54. In addition, a few studies have reported on mitochondrial haplogroups and population genetics related to PCOS susceptibility. Mitochondrial haplogroups are genetic lineages classified based on variations in mtDNA sequences, reflecting the evolutionary history of human maternal inheritance. Recent reports have indicated that specific mitochondrial haplogroups may serve as genetic backgrounds, interacting with functional mutations to participate in the pathogenesis of PCOS. Leng et al. reported a case of a Chinese PCOS patient. The researchers conducted whole mitochondrial genome sequencing on this patient and found that she carried a set of polymorphic variations specific to mitochondrial haplogroup F2. Additionally, the patient had two homoplasmy point mutations: the ND5 T12338C mutation and the tRNASer (UCN) C7492T mutation. As the genetic background of the patient, haplogroup F2 itself may not directly cause disease, but when combined with the above two functional mutations, it may synergistically lead to mitochondrial dysfunction and increase the risk of PCOS 55. Another study by the same group reported a three-generation family with a maternally inherited pattern. Multiple members of this family suffered from metabolic syndrome, and a female in the third generation also presented with PCOS. Mitochondrial gene analysis of the family members revealed that all affected individuals carried a set of genetic variations belonging to the East Asian mitochondrial haplogroup B4b1c. Moreover, the researchers discovered three homoplasmy mitochondrial tRNA mutations, which led to significant mitochondrial dysfunction 56. mtDNA is maternally inherited in most animals and is characterized by a rapid evolutionary rate and haploid inheritance, making it an important tool in population genetics research. The genetic variations, distribution, dynamics and evolutionary significance of mtDNA within a population are the main contents of mitochondrial population genetics. Based on the analysis of mtDNA haplotypes, it has been widely used to assess the genetic structure, gene flow, historical dynamics and systematic geographical information of populations 57. In a study of the Chinese population, Chen et al. identified that certain mtDNA D-loop mutations (G207A, 16036GGins, 16049Gins) and haplotype A15 confer protection against PCOS, and further demonstrated that PCOS patients have significantly higher mtDNA copy numbers than controls 58. A recent cross-ethnic study by Nawaz et al. analyzed the mitochondrial transfer RNA (mt-tRNA) genes in 64 Pakistani patients with PCOS and compared the identified mutations with patients from other ethnic groups. Eight variants in five mt-tRNA genes were found, most of which were novel and occurred in highly conserved nucleotides of tRNA. The study revealed that certain mt-tRNA genes carrying PCOS-associated mutations may be specific to particular ethnic populations 59.

#### 2.3.2 Mitochondrial dynamics

Mitochondria are extremely dynamic organelles that undergo constant cycles of fission and fusion, changing their morphology, size, and distribution. This process, known as mitochondrial dynamics, is crucial for maintaining cellular homeostasis. Fusion involves the merging process of both the inner and outer mitochondrial membranes, enhancing the resilience of the mitochondrial network and facilitating the exchange of mitochondrial contents. The inner mitochondrial membrane fusion protein optic atrophy 1 (OPA1) and the outer mitochondrial membrane fusion proteins mitofusin 1 and 2 (MFN1/2) co-regulate this process 60, 61. Conversely, mitochondrial fission partitions the tubular mitochondrial network into smaller fragments, which aid in the removal of depolarized mitochondria via mitophagy. The process of fission is primarily regulated by dynamin-related protein 1 (DRP1). After phosphorylation-induced activation, DRP1 translocates to the outer mitochondrial membrane and interacts with mitochondrial fission factor (MFF) to drive the fission process. Under normal physiological conditions, mitochondrial fusion and fission are tightly balanced to maintain mitochondrial integrity. Disruption of this equilibrium impairs mitochondrial function and contributes to disease 62.

To date, there have been few studies on dynamic mitochondrial changes in PCOS. Salehi et al. found that mitochondrial fission increased and protein levels of DRP1 and phospho-DRP1 (Ser616) were upregulated in a dihydrotestosterone (DHT)-induced rat model of PCOS 63. Li et al. also reported that the fission cofactors Fis1 and MFF were significantly increased in this model. Although DRP1 expression level did not change, activated phospho-DRP1 (Ser616) increased markedly, whereas the inactivated phospho-DRP1 (Ser637) protein expression level decreased significantly. The addition of the DRP1 inhibitor Mdivi-1 has the potential to reverse this process. The imbalance in DRP1 phosphorylation might have been involved in the mechanism underlying increased mitochondrial fission 64. Another study has reported the effect of mitochondrial fusion on PCOS and found that MFN2 protein expression levels were decreased in oocytes and granulosa cells of a PCOS model 21. However, a clinical study has demonstrated that in ovarian granulosa cells of patients with hyperandrogenic PCOS, the mRNA expression of mitochondrial fusion genes mitoguardin 1 and 2 was significantly upregulated, with expression levels positively correlated with serum testosterone concentrations 65. HA may enhance compensatory mitochondrial fusion, but evidence from animal models indicates that prolonged androgen exposure may ultimately lead to a predominance of fission and impairment of the fusion process. The reduction in mitochondrial fusion and the concomitant increase in mitochondrial fission exacerbate mitochondrial dysfunction, thereby promoting elevated levels of mitophagy 66.

#### 2.3.3 Mitophagy

**Figure 1:**
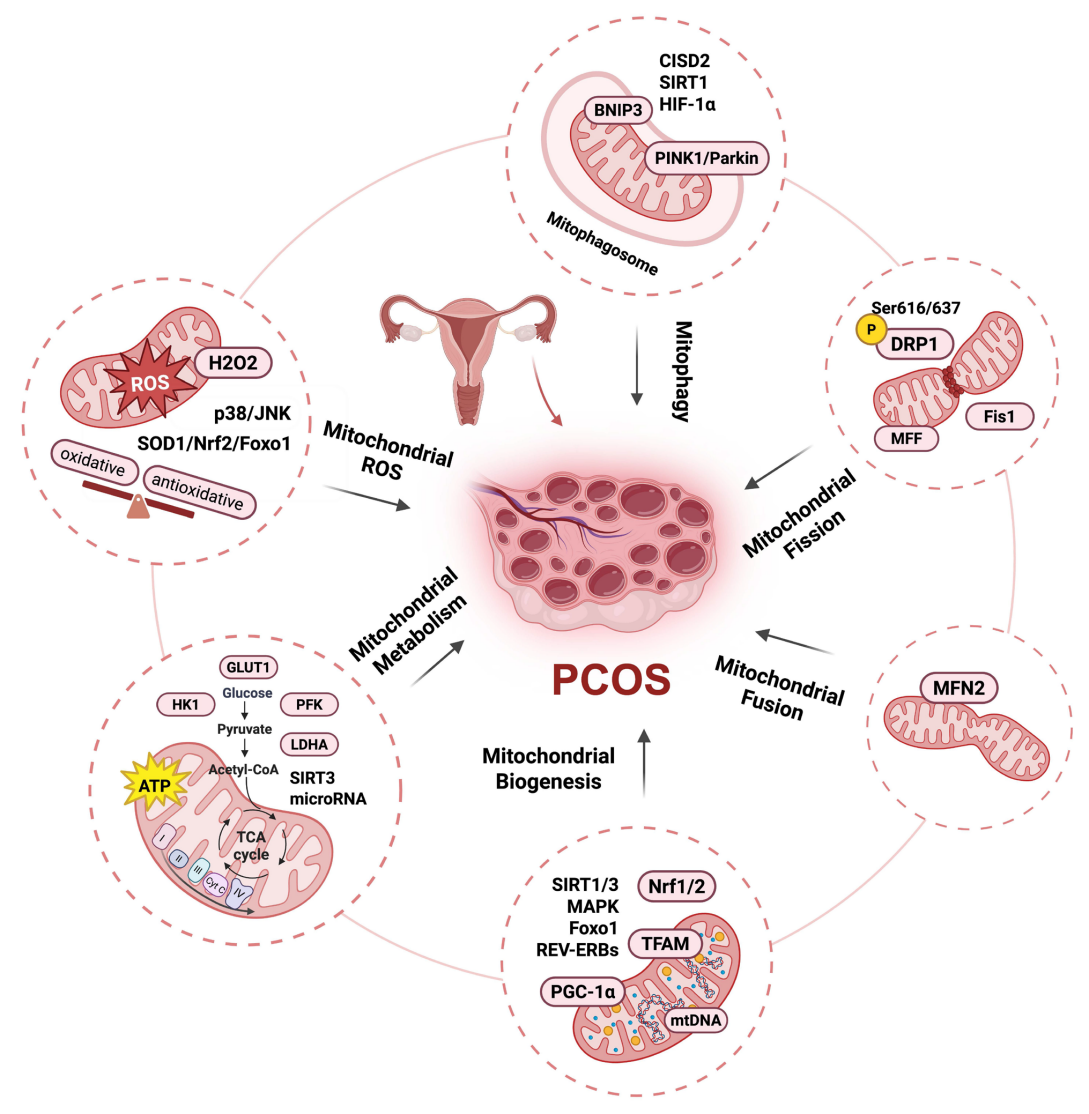
Mitochondria as a signaling hub in PCOS. Mitochondrial damage is primarily associated with mitochondrial oxidative stress, metabolic dysfunction, and quality control disorders. Clinical and basic studies show that PCOS exhibits an oxidative and antioxidative stress responses imbalance: the level of ROS and H_2_O_2_ emissions increase, accompanied by a decrease in oxygen consumption and GSH levels, which may be alleviated by metformin. Mitochondria-ROS-SOD1/Nrf2, Nrf2-Foxo1-ROS, and ROS/p38/JNK pathways have been reported in PCOS models. PCOS patients also exhibit reduced ATP content and decreased glycolysis that could by regulated by follicular fluid-derived exosomal miR-143-3p/miR-155-5p; glycolysis-associated gene level downregulation including GLUT1, LDHA, PFK, and HK1; and decreased mitochondrial oxidative phosphorylation levels regulated by SIRT3. Mitochondrial quality control disorder in PCOS including mitochondrial biogenesis decreases regulation by PGC-1α, TFAM, SIRT1/3, Foxo1, REV-ERBs, and MAPK; mtDNA copy number decreases; mitochondrial fission increase regulated by DRP1, p-DRP1 (Ser616/637), Fis1, and MFF; mitochondrial fusion decrease regulated by MFN2; mitophagy increase regulated by PINK/Parkin pathway, CISD2, and SIRT1, and decrease regulated by HIF-1α/BNIP3 pathway. This figure was created by Biorender (https://biorender.com/).

Mitophagy is a selective cellular process by which damaged or dysfunctional mitochondria are removed. This process involves the sequestration of mitochondria by autophagosomes and their subsequent fusion with lysosomes, and is primarily mediated by the PINK1-Parkin pathway. Mitophagy can also occur via non-ubiquitination-dependent pathways, such as the BCL2/adenovirus E1B interacting protein 3-like (BNIP3L) or FUN14 domain containing 1 (FUNDC1) pathway 67, 68. Current research indicates that mitophagy in PCOS does not simply exhibit unidirectional enhancement or reduction but rather exists in a state of imbalance. This imbalance is a crucial factor contributing to mitochondrial dysfunction in granulosa cells and ovarian tissues. Mitophagy-associated proteins PTEN-induced kinase 1 (PINK) and Parkin (an E3 ubiquitin ligase) are significantly upregulated in the granulosa cells of PCOS patients and can be regulated by the CDGSH iron sulfur domain 2 (CISD2) pathway. CISD2 activation can inhibit mitophagy via the PINK/Parkin pathway and the binding of mitochondria to lysosomes 69. Yi et al. also showed that Parkin and PINK1 protein levels were upregulated and that this upregulation could be alleviated by the addition of melatonin via activation of the SIRT1 pathway 70. However, some studies have demonstrated that mitophagy is impeded in PCOS. In the granulosa cells of patients with PCOS and in letrozole-induced rat models, the HIF-1α/BNIP3 mediated mitophagy is suppressed, resulting in a decrease in the clearance capacity of damaged mitochondria and accumulation of ROS, thereby inducing mitochondrial dysfunction 71. Evidence from studies on PCOS demonstrate the role of mitochondria as a signaling hub in PCOS, as illustrated in Figure 1.

## 3. Ferroptosis and mitochondrial dysfunction in PCOS

### 3.1 Ferroptosis and ovarian follicle development

In 2012, Stockwell discovered ferroptosis, a type of programmed cell death caused by iron-dependent lipid peroxidation. Unlike apoptosis and other types of cell death, ferroptosis is characterized by shrunken and dense mitochondria, rounding and swelling of cells, cell rupture with intact nuclei, and chromatin condensation 72. Ferroptosis is regulated by various cellular metabolic processes, including redox equilibrium, iron homeostasis, mitochondrial function, and glucose, lipid, and amino acid metabolism. Interconnected processes such as lipid metabolism, ROS biology, and iron control contribute to its initiation and progression 73. ROS produced by mitochondria lead to lipid peroxidation, resulting in irreparable damage to the cell membrane and ferroptosis.

Iron plays an important role in regulating ferroptosis sensitivity. The Fenton reaction generates free radicals that react with phospholipid-containing polyunsaturated fatty acid (PUFA-PL) chains, resulting in the formation of phospholipid hydroperoxides (PLOOHs), a hallmark of ferroptosis 74. Glutathione peroxidase 4 (GPX4) is the most important enzyme for suppressing ferroptosis. The system XC-activation, a cystine/glutamate antiporter for amino acid transport, causes extracellular cystine to be carried within the cell, intracellular glutamate is transported outside the cell. Glutathione, produced by cysteine, converts PLOOHs to phospholipid alcohols (PLOHs) via stimulating GPX4 to inhibit cell ferroptosis 75. Ferroptosis has been indicated in various pathophysiological processes such as immunity, aging, cardiovascular diseases, neurodegenerative diseases, endocrine metabolic diseases, and cancer 76.

Studies have shown that ferroptosis is related to the growth and development of ovarian follicles. A minor quantity of iron is innocuous to the ovaries, however, excessive iron can impair ovarian hormone production and follicle development. This significantly affects the endocrine system and fertility of female mice, potentially leading to infertility. Moreover, the ovarian reserve function of their offspring may also be genetically influenced 77. Ferritin can up-regulate the expression of NF-κB and inducible nitric oxide synthase (iNOS) in rat ovaries and down-regulate the expression of GPX4, leading to a decrease in estrogen levels. Excessive iron can also cause iron-dependent inflammatory and oxidative stress responses, resulting in ovarian damage and dysfunction 78. Under conditions of obesity, ferroptosis can be activated and contribute to the depletion of primordial follicles. Studies have shown that during the primordial to primary transition stage of follicles in obese mice, ferroptosis and cellular oxygen-related signaling pathways are significantly enriched. In the ovaries of obese mice, the depletion of primordial follicles increases, fat deposition around follicles is higher, and granulosa cell proliferation is enhanced 79. Ferroptosis also assumes a significant role in the process of ovarian aging. Research has indicated that in granulosa cells of patients with diminished ovarian reserve and advanced age, GPX4 expression is downregulated and GSH levels are decreased, suggesting that ferroptosis participates in the process of ovarian aging. *In vitro* experiments have revealed that ferroptosis inducers impede granulosa cell growth by downregulating GPX4, whereas ferroptosis inhibitors can upregulate GPX4 expression and reverse this inhibitory effect. In mouse models, GPX4 expression is reduced in the oocytes of aged mice, and treatment with ferrostatin-1 (Fer-1) can enhance the number and quality of retrieved oocytes 80.

### 3.2 Ferroptosis and PCOS

Kim et al. found that metabolic abnormalities such as hepatocellular adenoma with iron-related disease (HAIR) are associated with elevated circulating iron levels 81. Higher serum ferritin levels have also been reported in PCOS women, indicating iron overload 82. Zhang et al. reported an association between ferroptosis and PCOS 83. In their study, maternal rats treated with 5α-DHT and insulin exhibited reduced levels of GPX4 and glutathione, as well as upregulated levels of malondialdehyde and glutathione + glutathione disulfide in the gravid uterus, and these compounds may participate in regulating ferroptosis. They also observed aberrant expression of ferroptosis-associated genes including Acsl4, Slc7a11, Tfrc, and Gclc, as well as increased iron deposition. Ferroptosis is mediated by the MAPK signaling system, which is triggered in the gravid uterus and includes extracellular signal-regulated kinase (ERK), p38, and c-Jun NH2-terminal kinase (JNK).

In addition to ferroptosis in the uterus, Hu et al. observed placental ferroptosis in a PCOS model 84. After administration of the antioxidant N-acetylcysteine, GPX4 protein levels increased, and ferroptosis in the gravid uterus and placenta was reversed. Administration of N-acetylcysteine could be a viable therapeutic approach for PCOS. Granulosa cells of the ovary also undergo ferroptosis in patients with PCOS and may be regulated by the circRHBG/miR-515/SLC7A11 axis 85. Tan et al. reported that miR-93-5p could also regulate ferroptosis in granulosa cells by regulating the NF-κB signaling pathway 86. Additionally, a recent study discovered that ferroptosis was elevated in PCOS women ovaries and in rat models induced by DHEA. The addition of Fer-1, a ferroptosis inhibitor, was found to alleviate a cluster of PCOS traits, including HA and ovulatory dysfunction. HA is regarded as a key factor triggering ferroptosis. In granulosa cells of PCOS women and ovaries of PCOS rats, this study found that concentrations of Fe^2+^ and malondialdehyde were increased, protein levels of nuclear receptor coactivator 4 (NCOA4) were upregulated, and GPX4 and ferritin heavy chain 1 (FTH1) protein levels were downregulated 87. Thus, activation of NOCA4-dependent ferritinophagy may be significant processes underlying ferroptosis in ovarian granulosa cells. Bioinformatics analyses revealed 14 differentially expressed genes in granulosa cells between PCOS patients and non-PCOS women, including LPIN1, BNIP3, DDIT4, ATF3, NOS2, NQO1, SLC2A6, and SLC2A1, which were enriched in mitochondrial outer membrane, ROS metabolic processes, and antioxidant activity, as ferroptosis 88. Although current evidence consistently indicates elevated levels of ferroptosis in PCOS, heterogeneity across study populations may exist, and these findings do not imply a universal increase in all individuals with PCOS.

### 3.3 Ferroptosis regulated by mitochondria in PCOS

Mitochondria play a key role in regulating ferroptosis 89, 90. Ferroptosis causes dramatic morphological changes in the mitochondria, including mitochondrial shrinkage, fragmentation, and cristae enlargement 91. Zhang et al. observed shrunken mitochondria with electron-dense cristae and increased levels of the mitochondria-encoded gene Dpp4, which can induce ferroptosis in the uteri of rats 83. The most significant mechanism of ferroptosis in mitochondria is the production of ROS. Mitochondrial ROS production induces ferroptosis by promoting lipid peroxidation 92. An *in vitro* study discovered the role of ROS signaling in ferroptosis triggered by ferric ammonium citrate (FAC) in KGN cells. FAC promotes the expression of NADPH oxidase 1 (NOX1) signaling, which is a vital regulator of oxidative stress. In this work, inhibition of NOX1 reversed the effects of FAC, increased GPX4 levels, and reduced Fe^2+^ release. This study also found that the transferrin receptor can increase iron level, promote ROS release, increase ACSL4 levels, activate mitophagy via the PINK1 pathway, and induce lipid peroxidation, indicating the role of the NOX1/PINK1/ACSL4 pathway in PCOS 93. Li et al. also reported the role of ROS in PCOS ferroptosis. Moreover, they found that treatment with baicalein, a flavonoid compound from the Lamiaceae family, could inhibit oxidative stress and ameliorate PCOS by suppressing chronic inflammation and lipid peroxidation and by modulating mitochondrial functions through regulation of glutathione peroxidase and the FTH1 signaling pathway to restrain ferroptosis in KGN cells 94. Another recently published study reported that n-3 PUFA dramatically reduces mitochondrial function in PCOS granulosa cells, resulting in a shift from high to low mitochondrial membrane potential. n-3 PUFA may also lead to mitochondrial sequestration and enhance the density of bilayer structures in KGN cells, which is correlated with the ultrastructure of ferroptosis 95.

**Figure 2:**
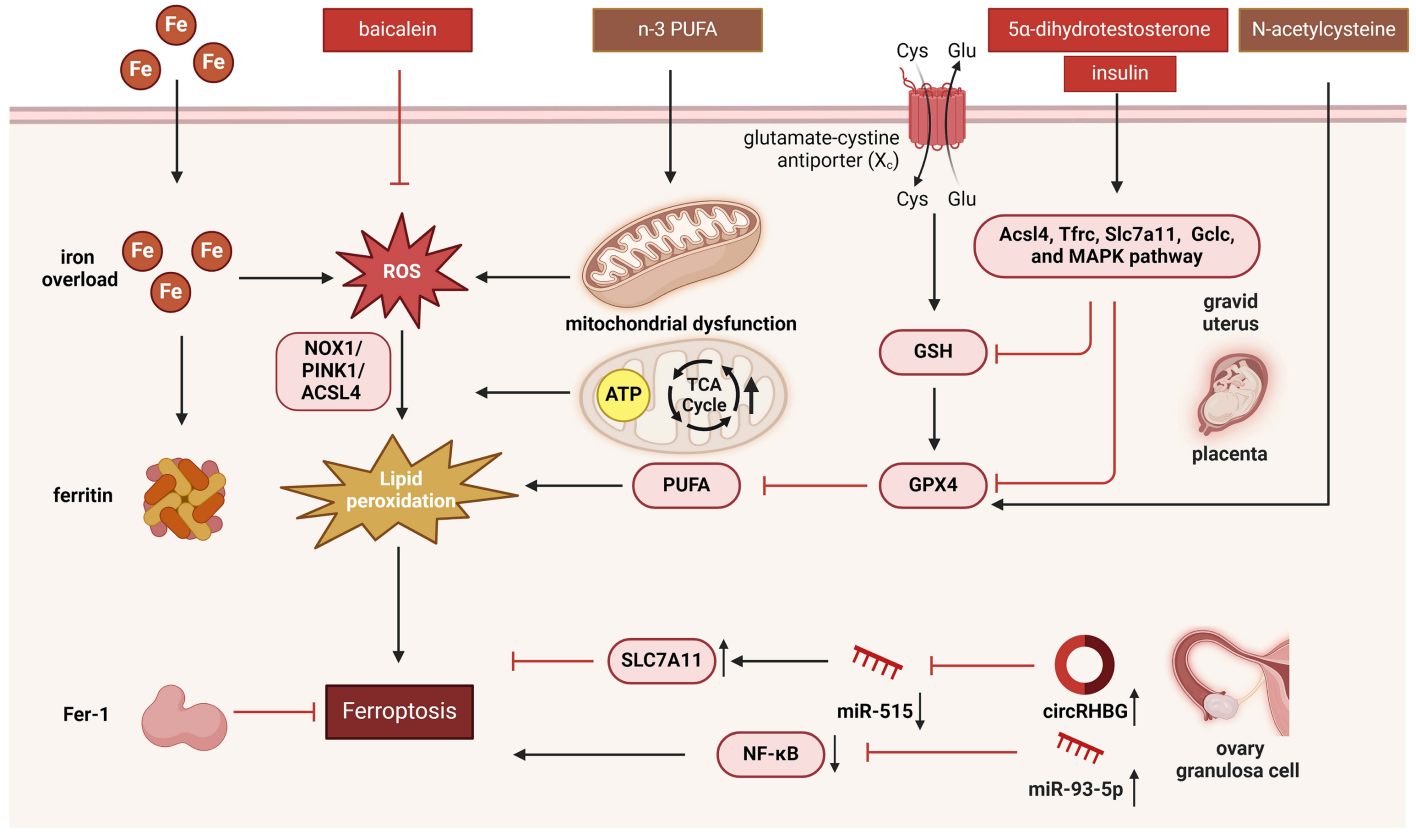
The mechanism of ferroptosis regulated by mitochondria in PCOS. Ferroptosis is characterized by shrunken and dense mitochondria driven by iron-dependent lipid peroxidation, and can be regulated by mitochondria. Ferroptosis in PCOS can be regulated by mitochondria via mitochondrial ROS regulated by NOX1, glutathione peroxidase, and FTH1; mitochondria-encoded gene Dpp4; mitophagy regulated by the NOX1/PINK1/ACSL4 pathway; TCA cycle and ATP production; p53 pathway and Nrf2-regulated iron and lipid metabolism pathways; and the mitochondrial voltage-dependent anion channel pathway. This figure was created by Biorender (https://biorender.com/).

Mitochondria are the primary organelles responsible for ATP production. AMP-activated protein kinase could inhibit the synthesis of certain PUFAs and ferroptosis by phosphorylating and suppressing acetyl-CoA carboxylases 96. Mitochondria also perform biosynthetic functions during cellular metabolism. The TCA cycle and several anaplerotic processes that repel the TCA cycle, such as glutaminolysis, are located in the mitochondria. Inhibition of glutaminolysis or glutamine deficiency significantly reduces ferroptosis. The mechanisms underlying the regulation of ferroptosis by the TCA cycle are likely related to electron transport and fatty acid production 97. Ferroptosis can also be influenced by other mitochondrial pathways, including those involved in sulfur transfer, p53, Nrf2-regulated iron and lipid metabolism, and the mitochondrial voltage-dependent anion channel pathways 98, 99. Changes in the regulation of mitochondrial dynamics also affect mitochondrial function and consequently induce ferroptosis 100. Collectively, these findings establish mitochondria as central hubs in the regulation of ferroptosis. On one hand, mitochondrial damage serves as both a trigger and a consequence of ferroptotic cell death. On the other hand, key ferroptosis regulators are intrinsically linked to mitochondrial metabolism. Notably, interventions such as baicalein and n-3 PUFA appear to ameliorate PCOS phenotypes by restoring mitochondrial function and inhibiting ferroptosis 101. The regulation of mitochondria and the underlying mechanisms remain incompletely understood and warrant further investigation. The mechanism of mitochondrial regulation of ferroptosis in PCOS is presented in Figure 2.

## 4. The inflammasome and mitochondrial dysfunction in PCOS

### 4.1 NLRP3 inflammasome and PCOS

PCOS is associated with a chronic inflammatory status 102. Under pathological conditions, oocyte development may be disturbed, resulting in follicular atresia and ovulatory dysfunction 103. Elevated levels of pro-inflammatory cytokines, C-reactive protein, and white blood cells have been reported in patients 104. Recently, inflammasomes have been demonstrated to be implicated in the pathophysiology of numerous inflammatory and metabolic illnesses 105.

As inflammasome-related cytokines, IL-18 and IL-1β are involved in the ovulatory process and follicular dynamics 106. Moreover, increased levels of IL-18 and IL-1β have been reported in the follicular microenvironment of PCOS women 107. Inflammasomes are intracellular multimeric complexes that recognize pathogen-associated and damage-associated molecular patterns, as first reported in 2002 108, 109. Inflammasome assembly induces the autoproteolytic processing of caspase-1, prompting the cleavage of its cellular substrates. Active caspase-1 cleaves the precursors of IL-18 and IL-1β to aid their maturation 110-113. Additionally, activated caspase-1 cleaves gasdermin D (GSDMD) into activated N-GSDMD, leading to cell membrane pore formation and cellular lysis, thereby inducing pyroptotic cell death and cytokine release, which are associated with various physiological processes 111, 114, 115. To elucidate the role of inflammasomes in the follicular microenvironment, it is crucial to explore the mechanisms underlying PCOS progression.

Several types of inflammasomes have been identified, of which the nod-like receptor (NLR) family pyrin domain-containing 3 (NLRP3) inflammasome is the most studied 116, 117. Intracellular and extracellular pathogenic signals stimulate the assembly and activation of the inflammasome complex. It has been documented those certain ionic signals, including ATP, lysosomal rupture, ROS, K^+^ efflux, Ca^2+^ signaling, and mitochondrial malfunction, activate the NLRP3 inflammasome 118. Moreover, abnormal metabolic status is related to the activation of NLRP3 inflammasomes. NLRP3 activation is implicated in steroidogenesis, oocyte maturation, autophagy, and apoptosis 119. HA is a characteristic feature of PCOS. A study found that hyperandrogen levels increase ROS production in ovarian cells by activating NLRP3 in PCOS rats 120. Higher levels of NLRP3 inflammasomes have been reported in the peripheral blood mononuclear cells and granulosa cells of women with PCOS than in the control group 119, 121. Liu et al. detailed the addition of formononetin *in vivo* and *in vitro* by inhibiting inflammation, apoptosis, and oxidative stress caused by the NLRP3 inflammasome 122. Li et al. observed that the gut microbial metabolite indole-3-propionic acid (IPA) alleviates PCOS in mice by regulating the aryl hydrocarbon receptor (AhR)/NLRP3 axis 123. A recent systematic review and meta-analysis also indicated that NLRP3 is upregulated in patients with PCOS and animal models 124. Several free fatty acid levels are significantly associated with mature IL-18 in follicular fluids, and oleic acid treatment activates inflammasome signaling in KGN cells 125. In addition, Liu et al. stimulated KGN cells with the follicular fluid from women with PCOS and found that it activated NLRP3 inflammasomes, the NF-κB pathway, and impaired mitochondria structure and function in KGN cells were found 107.

Abnormal metabolic status is associated with the activation of the NLRP3 inflammasome. Metabolic pathway alterations occur during macrophage activation, indicating that different metabolic processes lead to different inflammasome fates, including the regulatory effects of glycolytic enzymes, TCA cycle metabolites, and lipid pathways on inflammasomes. Taken together, the altered follicular microenvironment in PCOS induces inflammatory stress in oocytes and the surrounding granulosa cells. Several studies have demonstrated the involvement of inflammasome-dependent pathways and pyroptosis in ovarian dysfunction. For instance, exposure to the endocrine-disrupting substance di-(2-ethylhexyl) phthalate (DEHP) can cause reproductive system dysfunction. Sun et al. discovered a novel mechanism by which DEHP induces pyroptosis in ovarian granulosa cells via the SLC39A5/NF-κB/NLRP3 axis, thereby impairing ovarian function 126.

### 4.2 GSDMD-dependent pyroptosis and PCOS

GSDMD-dependent macrophage pyroptosis regulates various physiological processes involved in inflammation 127. Ovarian granulosa cell survival is essential for PCOS development and progression. Cellular pyroptosis occurs in PCOS and is associated with inflammasome activation. Wang et al. demonstrated that high androgen levels stimulate chronic low-grade inflammation in the ovaries of PCOS mice by activating the NLRP3 inflammasome and induce follicular dysfunction, ovarian granulosa cell pyroptosis, and ovarian interstitial cell fibrosis 128. Huang et al. reported that GSDMD-dependent macrophage pyroptosis impairs estrogen synthesis and induces apoptosis of granulosa cells in PCOS mice. Additionally, metformin treatment regulated gut microbiota abundance and inhibited macrophage pyroptosis in the ovaries, thus ameliorating PCOS 129.

However, the underlying molecular mechanism remains unclear. Cai et al. discovered that Wilms' tumor 1-associated protein (WTAP), a critical regulator of the RNA N6-methylase complex, is upregulated in granulosa cells, resulting in inflammasome overactivation. Overexpression of WTAP in granulosa cells stabilizes the mRNA expression of the inflammasome component apoptosis-associated speck-like protein (ASC), causing granulosa cell pyroptosis in PCOS 130. The role of miRNAs in regulating PCOS has been reported. For example, miR-29a-3p is downregulated in PCOS. Histone deacetylase 1 (HDAC1) inhibits granulosa cell pyroptosis in PCOS through deacetylation to regulate the H19/miR-29a-3p/NLRP3 axis 131. Wu et al. found that Guizhi Fuling Wan intervention upregulated the expression level of miR-29b-3p and downregulated the expression level of H19, thereby inhibiting granulosa cell autophagy in PCOS 132. Another study found that miR-1224-5p reduces NLRP3 inflammasome activation, IL-1β production, and NF-κB p65 nuclear translocation, lowering inflammation and preventing PCOS development 133. These findings improve our understanding of PCOS and provide potential new targets for its treatment.

### 4.3 Inflammasome regulated by mitochondria in PCOS

Mitochondria serve as organizational hubs for the assembly and activation of the NLRP3 inflammasome 134. Mitochondrial damage is implicated in the induction of inflammasome complexes. ROS is a common signal that activates the NLRP3 inflammasome 135. Impaired mitochondrial autophagy leads to prolonged mitochondrial ROS production 136. NLRP3 is directly activated by mitochondria-derived effector molecules. Zhong et al. found that mtDNA synthesis is crucial for NLRP3 signaling. The synthesis of oxidized mtDNA fragments requires mtDNA synthesis dependent on CDGSH iron sulfur domain 2 (CMPK2), a rate-limiting enzyme that provides deoxyribonucleotides for mtDNA synthesis 137. Oxidized mtDNA, released into the cytosol upon mitochondrial dysfunction, activates the NLRP3 inflammasome 138. The binding of mtDNA to the NLRP3 inflammasome complex is necessary for this activation.

**Figure 3:**
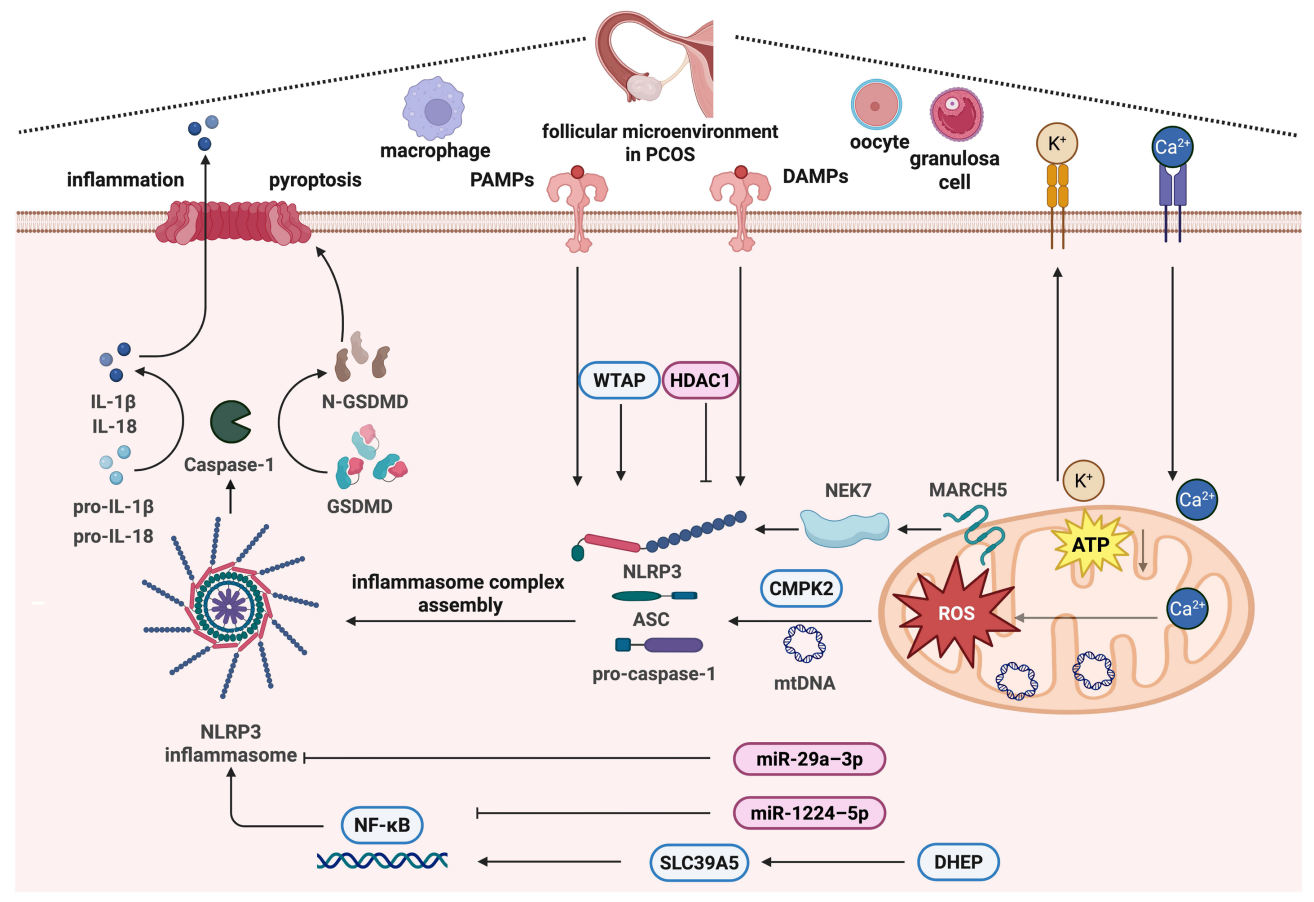
The mechanism of inflammasome activation regulated by mitochondria in PCOS. PCOS is known for its chronic inflammatory status, and inflammasome activation is an important mechanism, among which the NLRP3 inflammasome has been the most studied. NLRP3 inflammasome activation is regulated by mitochondria in PCOS via mitochondrial ROS regulated by Ca^2+^ signaling, the TCA cycle and ATP production, glucose and lipid metabolism pathways, mtDNA synthesis regulated by CMPK2, and mitochondrial quality control regulated by MARCH5 and NEK7. This figure was created by Biorender (https://biorender.com/).

Moreover, mtDNA release depends on inflammasome activation and mitochondrial ROS-dependent mechanisms 139. Mitochondria play a critical role in the activation of the NLRP3 inflammasome by binding NLRP3 to cardiolipin 140, and are the onset platform for the formation of the NLRP3 immune complex 19. It has been determined that membrane-associated RING-CH-type finger (MARCH) proteins of E3 ubiquitin ligases are important modulators of immunological responses. The outer membrane protein MARCH5 controls quality control and mitochondrial dynamics 141, 142. Mice with myeloid cell-specific MARCH5 conditional knockout (cKO) do not release IL-1β and IL-18, resulting in decreased mortality and inflammation during septic shock. Furthermore, the mitochondrial-resident MARCH5 E3 ligase is a possible regulator that initiates NEK7 binding to NLRP3 19. Taken together, these findings suggest that mitochondrial damage and dysfunction underlie abnormal follicular development in PCOS by driving the formation of NLRP3 inflammasomes and the subsequent induction of inflammatory signaling pathways (Figure 3).

## 5. ER stress and mitochondrial dysfunction in PCOS

### 5.1 ER stress and PCOS

The ER is the principal organelle responsible for protein biosynthesis, folding, secretion, lipid metabolism, and Ca^2+^ storage in eukaryotic cells 143, 144. Under endogenous or exogenous stimuli, ER homeostasis is disrupted, and persistently misfolded or unfolded proteins accumulate in the ER lumen, resulting in the induction of ER stress. Numerous physiological and pathological circumstances, including a breakdown of calcium homeostasis, disruption of redox homeostasis, pathogens, and protein variation, can cause ER stress 145, 146. ER stress and mitochondria interact closely through multiple mechanisms. ER stress sensors can directly trigger mitochondrial damage and inflammasome assembly. The ultrastructure of mitochondria-associated endoplasmic reticulum (MAM) is a core regulatory hub, and its dysfunction can cause calcium homeostasis imbalance, mitochondrial energy metabolism disorders and a vicious cycle of oxidative stress 147.

Cells initiate the unfolded protein response (UPR), an adaptive mechanism, to cope with ER stress. The three main ER sensors involved in initiating and regulating the UPR are inositol-requiring enzyme 1 (IRE1), protein kinase R (PKR)-like ER kinase (PERK), and activating transcription factor 6 (ATF6) 148, 149. Under ER stress, the three branches of the UPR (IRE1, PERK, and ATF6) activate several signaling cascades that take part in the regulation of protein synthesis, gene expression, and cell fate decisions such as apoptosis. The three signaling pathways not only have independent feedback loops but also cross-regulatory networks 150-152. However, the UPR plays a dual role in diseases. It can improve protein folding efficiency and promote ER-related protein degradation and autophagy, thereby eliminating misfolded or unfolded proteins to restore cellular homeostasis and promote cell survival under ER stress. When the ER returns to homeostasis, the UPR receives negative feedback and ER stress stops. On the other hand, when ER stress is prolonged or severe, it leads to cell death 153. ER stress and the UPR have been implicated in various diseases, including diabetes, obesity, neurodegenerative diseases, cancers, inflammatory diseases, and metabolic disorders 154.

ER stress has been implicated in follicular growth and maturation, follicular atresia, hormone production and secretion, and corpus luteum biogenesis 155-157. Genes and proteins involved in ER stress have been reported in granulosa cells, oocytes, cumulus-oocyte complexes, theca cells, the corpus luteum, and embryos 158. There is evidence that ER stress is induced in granulosa cells of women with PCOS and in PCOS mouse model, and that it plays a role in the interplay between inflammation and apoptosis. In human granulosa cells, thapsigargin and tunicamycin, which cause ER stress, raise the production of pro-fibrotic growth factors 159. A recent study reported increased inflammatory levels, pyroptosis, and ER stress sensor proteins in androgen-induced KGN cells. To investigate the function of ER stress in androgen-induced inflammation and pyroptosis, Xiang et al. treated KGN cells with tauroursodeoxycholic acid (an ER stress inhibitor) and found that suppressing ER stress reduced HA-induced inflammation and pyroptosis. Bioinformatics analyses have identified genes associated with ER stress in PCOS 160. These results suggest that ER stress may be critical in the imbalanced inflammatory microenvironment of PCOS 161.

### 5.2 Targeted ER stress therapy in PCOS

Recently, some studies have reported the potential value of inhibiting excessive ER stress in the treatment of PCOS. A randomized clinical trial found that ER stress exists in patients with PCOS and can be alleviated by the administration of astaxanthin (ASX). In this study, patients were classified into ASX or placebo groups, and the results demonstrated that ER stress in granulosa cells of PCOS women could be modulated by ASX. Additionally, the treatment group exhibited higher rates of high-quality oocytes, high-quality embryos, and oocyte maturation, whereas no significant differences were observed in oocyte number, fertilization rate, or fertility rate 162. Another randomized clinical trial by Jabarpour et al. also found that the ER stress-associated protein ATF6 was upregulated in women with PCOS, and the administration of ASX may benefit PCOS by modulating the ER stress-apoptotic pathway and reducing serum inflammatory marker levels 163. A further clinical study showed that resveratrol could improve symptoms of patients with PCOS by decreasing pro-inflammatory and ER stress markers 164. El-Saka et al. found that a PCOS group exhibited increased ER stress after administration of adrenomedullin (ADM) in a rat PCOS model. This can alleviate the disturbances of suppressing of PI3K/Akt1 and PPAR-γ pathways, the imbalance of the sex hormone profile, hyperglycemia, dyslipidemia, IR, increased profibrotic factors, and abnormal ovarian histopathological changes in the ovaries of the PCOS model by attenuating ER stress 165. Bai et al. showed that IL-18 binding protein (IL-18BP), a specific inhibitory receptor of IL-18, could reverse inflammation, fibrosis, and ER stress-related pathways in PCOS mouse ovaries, and that the level of mitochondrial ROS was also decreased 166. Electroacupuncture (EA) has also been reported that can alleviated PCOS by regulating ER stress *in vitro* and *in vivo* 167, 168. Furthermore, Peng et al. found that treatment with EA could improve mitochondrial dysfunction by facilitating the activity of complexes I and III in PCOS rats. The above results suggest that targeting and inhibiting ER stress holds a promising therapeutic prospect in PCOS.

### 5.3 ER stress regulated by mitochondria in PCOS

The pathogenesis of ER stress in PCOS is primarily associated with the UPR triggered by the IRE1, PERK, and ATF6 signaling pathways. Moreover, the ER and mitochondria are closely interconnected and can influence each other. Mitochondria also exhibit UPR after stress. A clinical study found that the UPR genes of the ER, including IREI, ATF6, XBP1, BIP, and CHOP, and the UPR genes of the mitochondria, including HSP10, HSP60, CLPP, and HSP40, were increased in granulosa cells of women with PCOS 169.

ER stress is closely associated with increased mitochondrial ROS levels. Clinical, *in vivo*, and *in vitro* studies have reported that PCOS causes ER stress and increases ROS level enhancement 170-172. The ER and mitochondria interact to ensure cell functional coordination through multiple contact sites, called the MAM, which are involved in the regulation of the intracellular microenvironment, especially in the exchange of ROS and Ca^2+^. The MAM is tightly connected to the outer membrane of the mitochondria, allowing Ca^2+^ transfer between the two organelles. ER stress often leads to Ca^2+^ flow from the ER to the mitochondria via MAMs, resulting in elevated mitochondrial ROS levels, which in turn intensify ER stress and Ca^2+^ release. This vicious cycle between ER stress and mitochondrial disorder promotes apoptosis 20, 147, 173, 174. Liao et al. reported that the mitochondrial fusion protein MFN2 regulates MAMs to affect PCOS oocyte development in a mouse model. The expression levels of proteins and genes of mitochondria-related proteins, IP3R, were downregulated in the MAMs of the PCOS model. In order for Ca^2+^ to be released from the ER into the cytoplasm, IP3R is a crucial Ca^2+^ release channel in the region. Together with glucose-regulated protein 75 (GRP75) and voltage-dependent anion channel 1 (VDAC1), it forms Ca^2+^ channel proteins that control Ca^2+^ transport between the mitochondria and the ER 21. Yuan et al. used a PCOS cell model to show that intracellular Ca^2+^ levels increase and induce ER stress 175. Malfunctioning MAM fail to effectively coordinate mitochondrial biogenesis, dynamics, and quality control, leading to a series of functional disorders, including reduced mitochondrial quantity, abnormal structure, insufficient ATP production, excessive ROS generation and decreased membrane potential. These defects ultimately impair the normal maturation and developmental potential of oocytes and promote the apoptosis of granulosa cells, collectively contributing to abnormal ovarian function and reduced fertility in PCOS 176.

**Figure 4:**
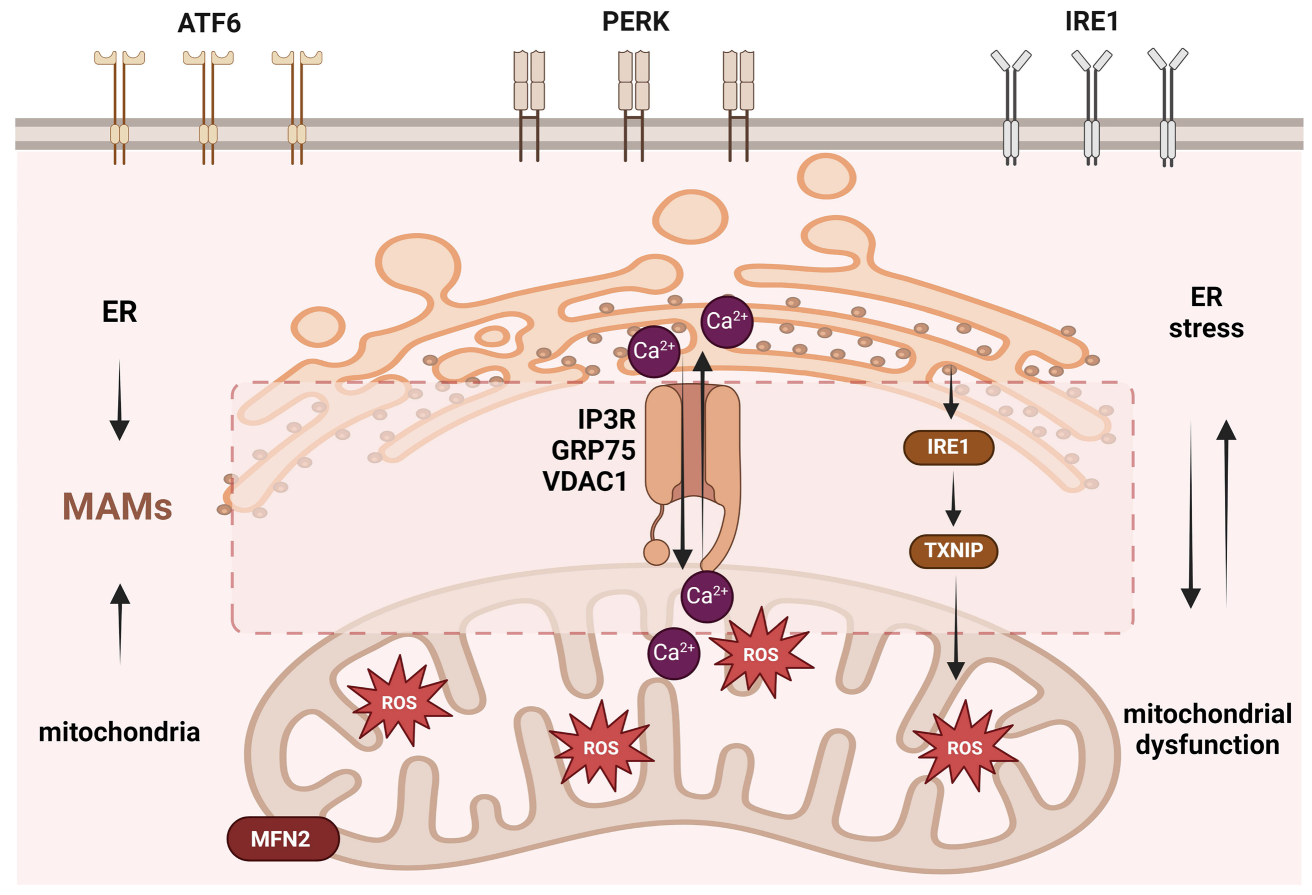
The mechanism of endoplasmic reticulum stress regulated by mitochondria in PCOS. ER stress is primarily induced by UPR regulation via the IRE1, PERK, and ATF6 pathways. ER stress in PCOS can be regulated by the mitochondria via mitochondrial ROS regulated by IL-18BP; Complex I and Complex III; and by mitochondrial UPR regulated by HSP60, HSP10, CLPP, and HSP40. In addition, the ER and mitochondria have a tightly connected structure called MAMs, which can regulate the exchange of ROS and Ca^2+^ and can be regulated by the mitochondrial fusion protein MFN2 and Ca^2+^ release channel protein IP3R. This figure was created by Biorender (https://biorender.com/).

PCOS is a complex process, and ER stress can also regulate ferroptosis and inflammasome activation. Ge et al. demonstrated that the granulosa cells from women with PCOS and from a mouse model exhibited ER stress and mediated ferroptosis in granulosa cells, leading to follicular dysfunction induced by HA. Inhibition of ER stress suppressed HA-induced ferroptosis 177. Weng et al. found that ROS production in the ovarian cells of a PCOS model induced ER stress and activated the IRE1-TXNIP/ROS-NLRP3 signaling pathway, increasing inflammatory levels 120. The regulation of MAMs may represent a key mechanism in these processes. MAMs control both Ca²⁺ transfer and ROS signaling, and dysregulation of Ca²⁺ and ROS is known to be closely associated with lipid peroxidation in ferroptosis and the assembly of the NLRP3 inflammasome. As a signaling hub at the interface of these pathways, MAMs likely serve as an important platform that integrates cellular stress, metabolism, and death signals. In ferroptosis, MAM-mediated Ca²⁺ release may exacerbate ER stress and promote the accumulation of lipid peroxidation products, while MAM-derived ROS can directly oxidize polyunsaturated fatty acids, thereby initiating or amplifying the ferroptosis cascade. Regarding the NLRP3 inflammasome, MAM integrity may influence mitochondrial ROS release and Ca²⁺-dependent inflammatory signaling, thereby modulating NLRP3 oligomerization and caspase-1 activation 178, 179. However, current studies on ER stress caused by mitochondrial dysfunction in PCOS are limited and lack of detailed mechanistic insight (Figure 4).

## 6. Conclusion and future perspectives

PCOS is a systemic, multifactorial, polygenic, metabolic, and inflammatory disease. In this review, we highlight the role of mitochondrial dysfunction in the pathogenesis of PCOS. We propose that three major pathological processes including ferroptosis, inflammasomes, and ER stress, may lead to ovarian dysfunction, metabolic abnormalities, and chronic inflammation in PCOS.

Approximately 10% of women of reproductive age worldwide have PCOS, but 70% of patients fail to be diagnosed early 180. Research advances have been made in the mechanism of mitochondrial damage, ferroptosis, inflammasome activation, and ER stress in PCOS. However, there are relatively limited reports on predictive indicators of PCOS, especially in the adolescent population. Mitochondria are sensitive organelles that often become abnormal in the early stages of disease, and early markers warrant further exploration and validation. Qasemi et al. reported the potency of cell-free mtDNA levels in follicular fluid as a biomarker for PCOS in women 181. Another clinical study reported the predictive value of the oxidative stress-associated biomarker 8-Isoprostane 182. However, the sample size of this study was small.

Currently, there is a lack of early predictive indicators and reference ranges that can be widely applied in clinical practice. For high-risk women of reproductive age who are obese or have diabetes, early detection and intervention based on appropriate predictive indicators could significantly reduce the incidence of PCOS and its adverse outcomes. This represents an important direction for future research on PCOS.

## Abbreviations

ACSL4: synthetase long-chain family member 4
ADM: adrenomedullin
AhR: aryl hydrocarbon receptor
ATF6: activating transcription factor 6
ATP: adenosine triphosphate
ASC: apoptosis-associated speck-like protein
ASX: astaxanthin
BNIP3L: the BCL2/adenovirus E1B interacting protein 3-like
CISD2: CDGSH iron sulfur domain 2
CLPP: caseinolytic protease P
CMPK2: cytidine/uridine monophosphate kinase 2
CoA: acyl-coenzyme A
DEHP: di-(2-ethylhexyl) phthalate
DHEA: dehydroepiandrosterone
DHT: dihydrotestosterone
Dpp4: dipeptidyl peptidase-4
DPP-IV: dipeptidyl peptidase-IV
DRP1: dynamin-related protein 1
EA: electroacupuncture
ER: endoplasmic reticulum
ERK: extracellular signal-regulated kinase
ETC: electron transport chain
FAC: ferric ammonium citrate
Fer-1: ferrostatin-1
Fis1: mitochondrial fission 1 protein
Foxo1: Forkhead Box Protein O1
FTH1: ferritin heavy chain 1
FUNDC1: FUN14 domain containing 1
GLUT1: glucose trans porter-1
GPX4: glutathione peroxidase 4
GRP75: Glucose-regulated protein 75
GSDMD: activated caspase-1 cleaves gasdermin D
GSH: glutathione
HA: Hyperandrogenism
IR: insulin resistance
HAIR: hepatocellular adenoma with iron-related disease
HDAC1: Histone deacetylase 1
HIF-1α: hypoxia-inducible factor-1 alpha
HK1: hexokinase 1
H_2_O_2_: hydrogen peroxide
HSP60: heat shock protein 60
IL-18BP: IL-18 binding protein
IP3: inositol 1,4,5-triphosphate receptor
IPA: indole-3-propionic acid
IRE1: inositol-requiring enzyme 1
JNK: c-Jun N-terminal kinase
LDHA: lactate dehydrogenase A
mt-tRNA: mitochondrial transfer RNA
MAMs: mitochondria-associated endoplasmic reticulum
MAPK: mitogen-activated protein kinase
MARCH5: mitochondrial ubiquitin ligase membrane-associated RING-CH
MFF: mitochondrial fission factor
MFN2: mitofusin-2
mtDNA: mitochondrial DNA
MQC: mitochondrial quality control
NCOA4: nuclear receptor coactivator 4
NEK7: NIMA-related kinase 7
NLRP3: NLR family pyrin domain containing 3
NOX1: NADPH oxidase 1
Nrf2: NF-E2-related factor 2
OPA1: optic atrophy 1
PCOS: polycystic ovary syndrome
PERK: protein kinase R (PKR)-like ER kinase
PFK: phosphofructokinase
PGC-1α: peroxisome proliferator-activated receptor gamma coactivator
PINK: PTEN-induced kinase
PLOHs: phospholipid alcohols
PLOOHs: phospholipid hydroperoxides
RBP4: retinol-binding protein 4
ROS: reactive oxygen species
SIRT1: Sirtuin 1
SIRT3: Sirtuin 3
SOD1: superoxide dismutase 1
TCA: tricarboxylic acid
TFAM: mitochondrial transcription factor A
UPR: unfolded protein response
VDAC1: voltage-dependent anion channel 1
WTAP: Wilms' tumor 1-associated protein
